# (*E*)-2-[2-(2-Thien­yl)vin­yl]-1*H*-1,3-benzimidazole

**DOI:** 10.1107/S1600536808006405

**Published:** 2008-05-03

**Authors:** Tian Hang, Qiong Ye

**Affiliations:** a Ordered Matter Science Research Center, Southeast University, Nanjing 210096, People’s Republic of China

## Abstract

In the title compound, C_13_H_10_N_2_S, the dihedral angle between the imidazole and thio­phene rings is 16.89 (19)°, and the double bond adopts an *E* configuration. In the crystal structure, N—H⋯N hydrogen bonds link the mol­ecules into rows along *b*. There is also evidence of weak C—H⋯S inter­actions.

## Related literature

For general background, see: Huang *et al.* (2003 ▶); Wang *et al.* (2005 ▶); Ye *et al.* (2006 ▶, 2007 ▶). For the crystal structures of related compounds, see: Ozbey *et al.* (1998 ▶); Li & Clarkson (2007 ▶).
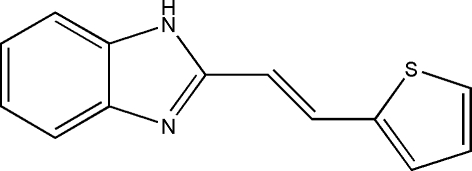


## Experimental

### 

#### Crystal data

C_13_H_10_N_2_S
*M* *_r_* = 226.06
Orthorhombic, 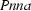

*a* = 12.239 (2) Å
*b* = 16.389 (3) Å
*c* = 11.487 (2) Å
*V* = 2304.1 (7) Å^3^
*Z* = 8
Mo *K*α radiation
μ = 0.25 mm^−1^
*T* = 293 (2) K
0.15 × 0.10 × 0.07 mm

#### Data collection

Rigaku Mercury2 diffractometer
Absorption correction: multi-scan (*CrystalClear*; Rigaku, 2005 ▶) *T*_min_ = 0.796, *T*_max_ = 1.000 (expected range = 0.782–0.983)
21849 measured reflections
2637 independent reflections
1360 reflections with *I* > 2σ(*I*)
*R*_int_ = 0.145

#### Refinement

*R*[*F* ^2^ > 2σ(*F* ^2^)] = 0.084
*wR*(*F* ^2^) = 0.214
*S* = 1.07
2637 reflections
145 parameters
1 restraint
H-atom parameters constrained
Δρ_max_ = 0.22 e Å^−3^
Δρ_min_ = −0.28 e Å^−3^

### 

Data collection: *CrystalClear* (Rigaku, 2005 ▶); cell refinement: *CrystalClear*; data reduction: *CrystalClear*; program(s) used to solve structure: *SHELXS97* (Sheldrick, 2008 ▶); program(s) used to refine structure: *SHELXL97* (Sheldrick, 2008 ▶); molecular graphics: *SHELXTL* (Sheldrick, 2008 ▶); software used to prepare material for publication: *SHELXTL*.

## Supplementary Material

Crystal structure: contains datablocks I, global. DOI: 10.1107/S1600536808006405/sj2460sup1.cif

Structure factors: contains datablocks I. DOI: 10.1107/S1600536808006405/sj2460Isup2.hkl

Additional supplementary materials:  crystallographic information; 3D view; checkCIF report

## Figures and Tables

**Table 1:** Hydrogen-bond geometry (Å, °)

*D*—H⋯*A*	*D*—H	H⋯*A*	*D*⋯*A*	*D*—H⋯*A*
C11—H11*A*⋯S1	0.93	2.76	3.161 (4)	107
N1—H1*B*⋯N1^i^	0.86	2.01	2.865 (6)	170
N2—H2*B*⋯N2^ii^	0.86	2.11	2.906 (5)	154

Symmetry codes: (i) 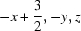; (ii) 
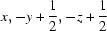
.

**Fig. 1.**
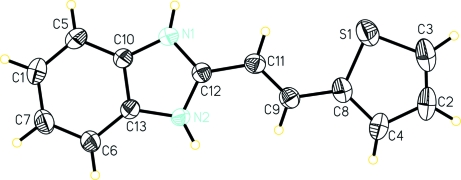
The molecular structure of the compound with the atomic numbering scheme. Displacement ellipsoids were drawn at the 30% probability level.

**Fig. 2.**
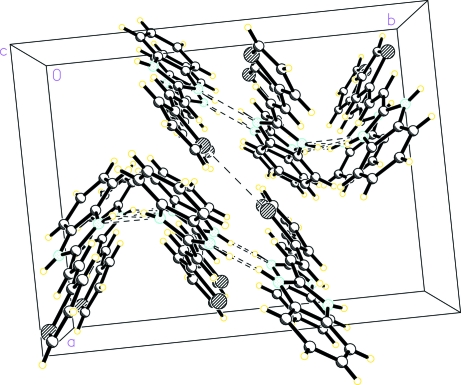
The crystal packing of 1 with hydrogen bonds drawn as dashed lines.

## supplementary crystallographic information

### Comment

It has been generally accepted that imidazole groups play an important role in coordination chemistry (Huang *et al.*, 2003). A flexible ligand readily induces coordination compounds to crystallize in non-centrosymmetric space groups, which makes it possible to investigate their interesting physical properties such as second harmonic generation, ferroelectric and piezoelectric properties (Wang *et al.*, 2005). As a continuation of our work in this field, (Ye *et al.*, 2006, 2007), we have synthesized the title compound, 1, Fig 1.

The title compound, C_12_H_10_N_2_S, was successfully prepared through the reaction between 2-methyl-1*H*-benzo[*d*]imidazole and thiophene-2-carbaldehyde. It adopts a *trans* configuration about the C9?C11 bond and the dihedral angle between the mean plane of the imidazole ring and thiophenyl ring is 16.89 (19)°. The crystal packing is dominated by N—H···N interactions linking the molecules into rows along b, Fig 2. There is also evidence of weak C—H···S interactions.

### Experimental

2-methyl-1*H*-benzo[*d*]imidazole (10 mmol, 1.32 g) and thiophene-2-carbaldehyde (45 mmol, 5.04 g) were reacted as a melt at 180°C with stirring for 18 h. Then 20 ml 2-propanol and 1.5 g oxalic acid were added to the reaction mixture, the solution filtered and the precipitate washed with copious quantities of boiling water. The pH was adjusted to 8–9 with ammonia to afford the title compound as a pale-yellow solid powder. Crystals suitable for single-crystal X-ray diffraction studies were obtained by slow evaporation of a solution in ethanol at room temperature over several days.

### Refinement

All carbon-bound H atoms were positioned geometrically, with C—H = 0.93 Å and included in the refinement as riding, with*U*_iso_(H) = 1.2*U*_eq_. The H atoms attached to N were found in the difference Fourier map and were subsequently treated as riding atoms, with N—H = 0.86 Å, and *U*_iso_(H) = 1.2*U*_eq_(N).

### Crystal data

**Table:**

C_13_H_10_N_2_S	*F*_000_ = 944
*M**_r_* = 226.06	*D*_x_ = 1.305 Mg m^−^^3^
Orthorhombic, *P**n**n**a*	Mo *K*α radiation λ = 0.71073 Å
Hall symbol: -P 2a 2bc	Cell parameters from 14820 reflections
*a* = 12.239 (2) Å	θ = 3.0–29.2º
*b* = 16.389 (3) Å	µ = 0.25 mm^−^^1^
*c* = 11.487 (2) Å	*T* = 293 (2) K
*V* = 2304.1 (7) Å^3^	Block, colorless
*Z* = 8	0.15 × 0.10 × 0.07 mm

### Data collection

**Table:**

Mercury2 (2x2 bin mode) diffractometer	2637 independent reflections
Radiation source: fine-focus sealed tube	1360 reflections with *I* > 2σ(*I*)
Monochromator: graphite	*R*_int_ = 0.145
Detector resolution: 13.6612 pixels mm^-1^	θ_max_ = 27.5º
*T* = 293(2) K	θ_min_ = 3.3º
CCD profile fitting scans	*h* = −15→15
Absorption correction: multi-scan(CrystalClear; Rigaku, 2005)	*k* = −21→21
*T*_min_ = 0.796, *T*_max_ = 1.000	*l* = −14→14
21849 measured reflections	

### Refinement

**Table:**

Refinement on *F*^2^	Secondary atom site location: difference Fourier map
Least-squares matrix: full	Hydrogen site location: inferred from neighbouring sites
*R*[*F*^2^ > 2σ(*F*^2^)] = 0.084	H-atom parameters constrained
*wR*(*F*^2^) = 0.214	*w* = 1/[σ^2^(*F*_o_^2^) + (0.081*P*)^2^ + 0.7151*P*] where *P* = (*F*_o_^2^ + 2*F*_c_^2^)/3
*S* = 1.07	(Δ/σ)_max_ < 0.001
2637 reflections	Δρ_max_ = 0.22 e Å^−^^3^
145 parameters	Δρ_min_ = −0.28 e Å^−^^3^
1 restraint	Extinction correction: none
Primary atom site location: structure-invariant direct methods	

### Special details

**Table:**

Geometry. All e.s.d.'s (except the e.s.d. in the dihedral angle between two l.s. planes) are estimated using the full covariance matrix. The cell e.s.d.'s are taken into account individually in the estimation of e.s.d.'s in distances, angles and torsion angles; correlations between e.s.d.'s in cell parameters are only used when they are defined by crystal symmetry. An approximate (isotropic) treatment of cell e.s.d.'s is used for estimating e.s.d.'s involving l.s. planes.
Refinement. Refinement of *F*^2^ against ALL reflections. The weighted *R*-factor *wR* and goodness of fit *S* are based on *F*^2^, conventional *R*-factors *R* are based on *F*, with *F* set to zero for negative *F*^2^. The threshold expression of *F*^2^ > σ(*F*^2^) is used only for calculating *R*-factors(gt) *etc*. and is not relevant to the choice of reflections for refinement. *R*-factors based on *F*^2^ are statistically about twice as large as those based on *F*, and *R*- factors based on ALL data will be even larger.

### Fractional atomic coordinates and isotropic or equivalent isotropic displacement parameters (Å^2^)

**Table:**

	*x*	*y*	*z*	*U*_iso_*/*U*_eq_	Occ. (<1)
S1	0.88515 (10)	0.06908 (7)	0.51647 (11)	0.0801 (5)	
N1	0.6821 (2)	0.07121 (18)	0.1000 (3)	0.0577 (9)	
H1B	0.7264	0.0309	0.0924	0.069*	0.50
N2	0.5945 (2)	0.17665 (16)	0.1790 (2)	0.0452 (7)	
H2B	0.5751	0.2134	0.2283	0.054*	0.50
C1	0.5111 (4)	0.1178 (3)	−0.1555 (4)	0.0824 (14)	
H1A	0.4956	0.1028	−0.2319	0.099*	
C2	0.8179 (4)	0.1317 (3)	0.7051 (4)	0.0898 (15)	
H2A	0.8136	0.1453	0.7836	0.108*	
C3	0.8987 (5)	0.0855 (3)	0.6610 (4)	0.0890 (16)	
H3A	0.9559	0.0647	0.7053	0.107*	
C4	0.7391 (3)	0.1583 (3)	0.6209 (3)	0.0653 (11)	
H4A	0.6790	0.1913	0.6364	0.078*	
C5	0.5901 (4)	0.0750 (3)	−0.0942 (4)	0.0753 (13)	
H5A	0.6278	0.0316	−0.1277	0.090*	
C6	0.4751 (3)	0.2076 (2)	0.0054 (3)	0.0604 (11)	
H6A	0.4376	0.2514	0.0381	0.073*	
C7	0.4546 (4)	0.1825 (3)	−0.1057 (4)	0.0720 (12)	
H7A	0.4015	0.2094	−0.1492	0.086*	
C8	0.7672 (3)	0.1264 (2)	0.5105 (3)	0.0581 (10)	
C9	0.7079 (3)	0.1380 (2)	0.4039 (3)	0.0532 (10)	
H9A	0.6461	0.1709	0.4074	0.064*	
C10	0.6101 (3)	0.0995 (2)	0.0186 (3)	0.0515 (9)	
C11	0.7328 (3)	0.1064 (2)	0.3007 (3)	0.0526 (10)	
H11A	0.7950	0.0740	0.2961	0.063*	
C12	0.6700 (3)	0.1188 (2)	0.1941 (3)	0.0479 (9)	
C13	0.5538 (3)	0.1655 (2)	0.0678 (3)	0.0461 (9)	

### Atomic displacement parameters (Å^2^)

**Table:**

	*U*^11^	*U*^22^	*U*^33^	*U*^12^	*U*^13^	*U*^23^
S1	0.0830 (9)	0.0654 (8)	0.0918 (10)	−0.0012 (6)	−0.0304 (7)	0.0032 (6)
N1	0.0607 (19)	0.0583 (19)	0.054 (2)	0.0158 (16)	−0.0072 (15)	−0.0140 (16)
N2	0.0532 (17)	0.0401 (16)	0.0422 (17)	0.0063 (13)	0.0015 (13)	−0.0027 (13)
C1	0.097 (3)	0.096 (4)	0.054 (3)	0.004 (3)	−0.017 (3)	−0.015 (3)
C2	0.113 (4)	0.106 (4)	0.050 (3)	−0.026 (3)	−0.023 (2)	0.013 (3)
C3	0.112 (4)	0.076 (3)	0.079 (4)	−0.023 (3)	−0.043 (3)	0.023 (3)
C4	0.070 (3)	0.081 (3)	0.045 (2)	−0.018 (2)	−0.0037 (18)	0.001 (2)
C5	0.083 (3)	0.079 (3)	0.064 (3)	0.018 (2)	−0.011 (2)	−0.028 (2)
C6	0.072 (3)	0.054 (2)	0.055 (2)	0.009 (2)	−0.009 (2)	0.001 (2)
C7	0.081 (3)	0.071 (3)	0.064 (3)	0.009 (2)	−0.021 (2)	0.007 (2)
C8	0.063 (2)	0.051 (2)	0.061 (3)	−0.0153 (19)	−0.014 (2)	0.0087 (19)
C9	0.055 (2)	0.049 (2)	0.056 (3)	−0.0009 (18)	−0.0023 (18)	0.0054 (18)
C10	0.053 (2)	0.051 (2)	0.051 (2)	0.0034 (18)	−0.0051 (18)	−0.0070 (18)
C11	0.051 (2)	0.048 (2)	0.059 (3)	0.0005 (17)	−0.0004 (19)	−0.0009 (18)
C12	0.048 (2)	0.049 (2)	0.047 (2)	−0.0011 (17)	0.0016 (17)	0.0022 (17)
C13	0.052 (2)	0.0418 (19)	0.044 (2)	−0.0023 (17)	0.0020 (17)	0.0001 (16)

### Geometric parameters (Å, °)

**Table:**

S1—C3	1.690 (5)	C4—C8	1.415 (5)
S1—C8	1.723 (4)	C4—H4A	0.9300
N1—C12	1.341 (4)	C5—C10	1.379 (5)
N1—C10	1.366 (4)	C5—H5A	0.9300
N1—H1B	0.8600	C6—C7	1.365 (5)
N2—C12	1.335 (4)	C6—C13	1.385 (5)
N2—C13	1.383 (4)	C6—H6A	0.9300
N2—H2B	0.8600	C7—H7A	0.9300
C1—C5	1.386 (6)	C8—C9	1.436 (5)
C1—C7	1.390 (6)	C9—C11	1.329 (5)
C1—H1A	0.9300	C9—H9A	0.9300
C2—C3	1.344 (6)	C10—C13	1.401 (5)
C2—C4	1.435 (6)	C11—C12	1.461 (4)
C2—H2A	0.9300	C11—H11A	0.9300
C3—H3A	0.9300		
			
C3—S1—C8	92.0 (3)	C7—C6—H6A	121.3
C12—N1—C10	106.4 (3)	C13—C6—H6A	121.3
C12—N1—H1B	126.8	C6—C7—C1	121.6 (4)
C10—N1—H1B	126.8	C6—C7—H7A	119.2
C12—N2—C13	106.0 (3)	C1—C7—H7A	119.2
C12—N2—H2B	127.0	C4—C8—C9	126.3 (4)
C13—N2—H2B	127.0	C4—C8—S1	111.7 (3)
C5—C1—C7	121.6 (4)	C9—C8—S1	122.0 (3)
C5—C1—H1A	119.2	C11—C9—C8	126.3 (4)
C7—C1—H1A	119.2	C11—C9—H9A	116.8
C3—C2—C4	114.3 (4)	C8—C9—H9A	116.8
C3—C2—H2A	122.8	N1—C10—C5	131.2 (4)
C4—C2—H2A	122.8	N1—C10—C13	107.7 (3)
C2—C3—S1	112.8 (4)	C5—C10—C13	121.1 (4)
C2—C3—H3A	123.6	C9—C11—C12	125.0 (3)
S1—C3—H3A	123.6	C9—C11—H11A	117.5
C8—C4—C2	109.2 (4)	C12—C11—H11A	117.5
C8—C4—H4A	125.4	N1—C12—N2	112.7 (3)
C2—C4—H4A	125.4	N1—C12—C11	122.5 (3)
C1—C5—C10	117.0 (4)	N2—C12—C11	124.9 (3)
C1—C5—H5A	121.5	N2—C13—C6	131.5 (3)
C10—C5—H5A	121.5	N2—C13—C10	107.3 (3)
C7—C6—C13	117.5 (4)	C6—C13—C10	121.2 (3)

### Hydrogen-bond geometry (Å, °)

**Table:**

*D*—H···*A*	*D*—H	H···*A*	*D*···*A*	*D*—H···*A*
C11—H11A···S1	0.93	2.76	3.161 (4)	107
N1—H1B···N1^i^	0.86	2.01	2.865 (6)	170
N2—H2B···N2^ii^	0.86	2.11	2.906 (5)	154

Symmetry codes: (i) −*x*+3/2, −*y*, *z*; (ii) *x*, −*y*+1/2, −*z*+1/2.
